# Treatment with CPAP in Elderly Patients with Obstructive Sleep Apnoea

**DOI:** 10.3390/jcm9020546

**Published:** 2020-02-17

**Authors:** Tomas Posadas, Grace Oscullo, Enrique Zaldívar, Alberto Garcia-Ortega, José Daniel Gómez-Olivas, Manuela Monteagudo, Miguel Angel Martínez-García

**Affiliations:** Pneumology Department, Hospital Universitario y Politécnico La Fe, 46015 Valencia, Spain; t.posadas21@gmail.com (T.P.); enrique.zaldivar.olmeda@gmail.com (E.Z.); albortgva@gmail.com (A.G.-O.); jdaniel365@hotmail.com (J.D.G.-O.); monteagudo_man@gva.es (M.M.)

**Keywords:** obstructive sleep apnoea, elderly, continuous positive airway pressure

## Abstract

The population pyramid is changing as a result of the ever-increasing life expectancy, which makes it crucial to acquire an in-depth understanding of the diseases that most often affect the elderly. Obstructive sleep apnoea (OSA) affects 15%–20% of the population aged over 65 years. Despite this prevalence, there have been very few specific studies on the management of OSA in this age group, even though over 60% of the patients aged over 65-70 years who attend sleep units with suspicion of OSA receive treatment with continuous positive airway pressure (CPAP), on the basis of an extrapolation of the positive results achieved by CPAP in clinical trials involving middle-aged males. However, the latter’s form of presentation, evolution and, probably, prognosis comparing with OSA are not the same as those of elderly patients. Recent clinical trials performed on an exclusive series of elderly patients have shed light on the possible role of CPAP treatment in elderly patients with OSA, but there are still many questions that need to be answered. The physiological increase in the number of sleep-related disorders with the passing of years, and the lack of validated diagnostic and therapeutic tools for this age group are probably the greatest obstacles to define, diagnose and treat OSA in the elderly.

## 1. Introduction

There is absolutely no doubt that the population pyramid is changing. Progressive medical advances and improved social conditions have been associated with increasing longevity, and so elderly people are acquiring a particularly important role in the field of healthcare, especially in the First World. One recent study concluded that people born in the industrialised world at the start of the 21st century will have a life expectancy of more than 100 years, with, more importantly, a good quality of life in most cases [1]. It is therefore essential to meet the growing demands of elderly patients who, quite rightly, expect diagnostic and therapeutic management comparable to that provided to younger people. Obviously, in this context, the diseases that most pressingly need further investigation are those that are most prevalent in, or have a particularly great impact on, the elderly, and those where our knowledge is lacking. Obstructive sleep apnoea (OSA) is a clear example of such a disease, as it illustrates both these tendencies: a high prevalence among the elderly, and little knowledge of its impact in this age group [2].

It is well recognised that the treatment of choice for the most serious or symptomatic forms of OSA is continuous positive airway pressure (CPAP). This treatment has proved successful in improving not only patients’ sleep-related symptoms (particularly daytime sleepiness) and quality of life, but also CPAP has a significant impact on the metabolic, cardiovascular and neurocognitive consequences of OSA [3]. However, most of the key studies that have established the criteria for the indication of CPAP were performed on middle-aged males [4,5], and their results have subsequently been extrapolated to other age groups and to women—even though it has been established that the clinical manifestations, and consequences of OSA vary with age and sex. For example, the apnoea-hypopnoea index (AHI), the objective parameter on which the diagnosis of OSA is usually based, increases physiologically with age [6,7,8,9]. Furthermore, the questionnaires and tests used to evaluate symptoms of OSA, such as daytime sleepiness, have not been validated in elderly patients and, similarly, it has not been demonstrated that CPAP treatment has the same positive effect on elderly patients as it does on younger ones. Despite this absence of evidence, one study undertaken in Spain, using data analysed from over 50,000 sleep studies carried out between 2002 and 2008 in 16 different sleep units, showed that one out of every four of these studies (24.3%) was performed on individuals aged over 65 years, and most of these were men (64.9%). More than 70% presented an AHI > 10 events/hour, and around 70% of this subgroup were treated with CPAP, a percentage that gradually increased with years, especially in elderly males [10]. These data confirm that, despite the scanty scientific evidence available on the diagnosis, impact and treatment of OSA in the elderly, this age group receives a great amount of healthcare related to this disorder, and even more so in recent years. This review will analyse the existing evidence on the impact of OSA on the elderly with a particular focus on the effect of CPAP treatment in elderly patients, and the future challenges inherent in this situation, in light of the increasing presence of elderly patients in sleep units. For this purpose, the authors conducted a systematic search in Pubmed using the following keywords in the title or abstract: (sleep apnea OR sleep apnoea OR sleep-disordered breathing OR CPAP OR Continuous positive airway pressure) AND (elderly OR older OR dwelling OR old).

## 2. Physiological Changes in Sleep with Age

Some epidemiological studies conclude that more than half of the population aged over 65 years live with some type of chronic sleep disorder [11]. Generally speaking, both the structure and the duration of sleep change with age. The most characteristic changes are an increase in the percentage of superficial sleep and a decrease in that of deep sleep, with little variation in the percentage of Rapid Eyes Movement (REM) sleep. These changes are accompanied by an overall decrease in the total sleep time and a reduction in its efficiency, as well as an increase in the number of arousals. These variations are not solely dependent on endogenous changes, however, as they are also influenced by lifestyle changes and external factors such as diet, physical activity and exposure to light that present themselves with the passing of time, as well as by chronic comorbidities associated with old age that affect sleep. These include: insomnia, cardiopulmonary and neurocognitive diseases, restless leg syndrome, arthropathies, the use of hypnotic medication and OSA [12,13]. 

It is well known that sleep-disordered breathing (SDB) becomes physiologically more common with age, due to a greater tendency towards upper airway collapse [12,13]. Table 1 illustrates some of these possible associated factors. The degree of pharyngeal collapsibility depends on the interaction of all these factors. It is thus more difficult for a clinician to decide how many of the events observed in an elderly patient with excessive SDB are pathological and how many are physiological, and, more importantly, whether this situation requires treatment or not.

## 3. OSA in The Elderly

The increased airway collapsibility derived from various factors physiologically associated with age explains the increased number of SDB cases observed. If we accept the diagnosis of OSA as the presence of an AHI ≥ 10 with a compatible clinical picture (daytime sleepiness, defined as a score >10 on the Epworth Sleepiness scale (ESS)), 20% of males and 15% of women aged over 65 years would satisfy these criteria [14]. It is important to clarify, however, that the ESS, which is habitually used to subjectively evaluate the degree of sleepiness in patients of all ages, has not been validated for use in the elderly. Moreover, there is a significant age-related increase in sleepiness that is not dependent on the presence of OSA (due to comorbidities, inactivity, obesity, psychotropic medication, cognitive alterations, etc.), and so a cut-off point of 10 on the AHI and EES could possibly fall within the physiological interval of most elderly people. 

The clinical picture for OSA in the elderly is probably different from the one observed in younger people. With respect to sleepiness, a key symptom for assessing the severity of OSA, there are substantial difficulties in establishing its existence, and more particularly its relationship with a possible OSA in elderly people for various reasons: the high prevalence of sleepiness unrelated to OSA, an elderly person’s different perception of this symptom by the patient, and the social perception of sleepiness as a normal symptom in the elderly [15,16]. Accordingly, many of the clinical and anthropometric data usually associated with OSA in middle-aged people seem to have a more limited predictive value of OSA in the elderly, both because they are less specific and because they are more difficult to measure or are subject to adaptive mechanisms. In contrast, however, the presence of OSA in an elderly person seems to be more closely associated with a series of clinical factors more typical of this age group, usually in the neurocognitive sphere (depressive symptoms, epileptic crises, glaucoma, unexplained nocturia, frequent falls, dementia) and, possibly, an excess of cardiovascular events [6,7,8,9], and so these always have to be taken into account, as they often represent forms of the disease’s presentation. 

In regards to diagnosis, a complete polysomnography (PSG) is currently the gold standard for all age groups. The great demand created in recent years by the increasing number of patients referred on suspicion of OSA, and some centres’ limited access to PSG, have resulted in a proliferation of devices that do not record neurophysiological variables. As a result, the test’s diagnostic effectiveness is reduced, although this drawback can be acceptable in certain circumstances. A complete PSG study is recommended, however, in patients with underlying cardiopulmonary disease, unstable sleep, an ingestion of psychotropic medication capable of modifying sleep architecture, the possibility of a diagnosis other than OSA or a clinical picture suggestive of OSA, despite a negative simplified sleep test. Nevertheless, a simplified test would be acceptable in some cases, in circumstances where access to PSG is impossible, and in comparatively younger elderly patients with a clinical picture suggestive of OSA without any significant cardiopulmonary comorbidities or epidemiological findings. If it is decided to use a simplified device, home studies can take on a particular relevance in the elderly, as they allow patients to relax in their everyday environment assuming an increase in invalid studies [2].

There are very few studies available with a sufficient level of scientific evidence on the impact of OSA in the elderly. The main problems probably lie in the fact that we do not know the AHI cut-off point that can be considered pathological, whether the AHI is the best means of identifying OSA in the elderly, or, finally, which clinical symptoms/signs should be taken into consideration. Some studies have shown that an excessive SDB or daytime sleepiness are capable of reducing elderly people’s quality of life [17,18,19]. In respect of cardiovascular findings, some authors have demonstrated that, even in patients aged over 65–70 years, the number of SDB and intermittent hypoxaemia correlates with an increase in low-grade systemic inflammation, which is known to be a cardiovascular risk factor [20]. Some clinical studies have observed that OSA causes (including in the elderly): excessive hypertension (especially at night), cardiac arrhythmias, poorer systolic cardiac function, and a higher incidence of stroke, but not, paradoxically, any greater incidence of coronary events [21,22]. In any case, these harmful effects of OSA are usually milder in elderly patients than in younger ones. This more limited effect on coronary blood flow was observed in a classic study by Lavie on mortality in 14,583 males aged between 20–95 years who were followed up for over 4.5 years. These authors found that an AHI > 30 was only linked to an excess of mortality (with respect to those with an AHI < 10) in men aged under 50 years, after adjustment for the body mass index, whereas, surprisingly, those aged over 70 years presented a lower mortality than men of their age in the general population [23]. Lavie postulated the existence of some kind of mechanism that protected elderly people from the consequences of apnoeas. This “hypoxic preconditioning” hypothesis supposes that survivors of OSA who reach an advanced age produce an increase in their coronary vascularization as a result of intermittent hypoxaemia that would protect them from coronary events and, therefore, from an excess of mortality due to this cause [23]. This finding does not seem to be corroborated, however, in the cerebral blood flow, which is governed by other regulatory mechanisms.

Finally, regarding the effects of OSA on various neurocognitive parameters and disorders in elderly people, the results in the literature are contradictory, as both the deterioration of these functions through age and the increase of incidents of neurocognitive disease in the elderly not related to OSA can sometimes be insurmountable confounders [24]. However, some studies have observed that sleep fragmentation and intermittent hypoxemia could negatively impact the progression of depression symptoms, dementia and Alzheimer’s disease [25,26,27]. 

## 4. CPAP Treatment in Elderly Patients with OSA

CPAP has been prescribed for 30 years, but barely any studies have demonstrated, with a sufficient level of scientific evidence, its effectiveness in an exclusive series of elderly people, even though in Spain, for example, one in every four sleep studies is performed on patients aged over 65 years, and over 70% of these are treated with CPAP [10]. Consequently, the decisions currently taken with respect to the elderly population are generally based on the extrapolated results of clinical trials on younger adults, or on a mixture of age groups that includes elderly patients but does not subject them to any specific analysis. In recent years, however, well designed randomised clinical trials (RCT) have yielded some interesting findings.

### 4.1. Randomised Clinical Trials

A systematic review of the RCT in the literature investigating the effect of CPAP in patients with OSA reveals a widespread absence of individuals aged over 60–70 years in these trials. Of the few that did include elderly patients, the most recent ones tend to focus on the effect of CPAP treatment on various aspects of cardiovascular disease, while those that examine the effectiveness of CPAP on symptoms associated with OSA are earlier publications. Overall, it could be said that CPAP affects the clinical and sleep-structure variables by producing a significant drop in the number of respiratory events and an improvement of the sleep architecture, with a reduction in phase I superficial sleep and an increase in the deep sleep phases. These alterations result in an improvement in the symptoms associated with OSA, particularly OSA-related sleepiness. The data on the effect of CPAP on neurocognitive variables are more controversial, as some studies have shown improvements after CPAP in variables such as memory, executive functions, cognitive processes and concentration but others have failed to do so. In this respect, the study by Chong et al. [27] is particularly noteworthy, as they found a significant improvement in sleepiness after six weeks of CPAP treatment in 39 patients with a diagnosis of probable Alzheimer’s disease and a mean age of 78 (SD: 7) years, with an age range of 53–91 years. These patients also presented a good tolerance of the device, at around 5 h/night. 

Inconsistency is also evident in the results concerning the effect of CPAP on the quality of life, as measured by SF-36, a general questionnaire on the quality of life, or the Functional Outcomes of Sleep Questionnaire (FOSQ), which specifically examines the impact of sleepiness. As we have seen, however, such inconsistency occurs within a general context of positive effects of CPAP on measurements of sleepiness. 

With regards to the analysis of the effect of CPAP on various cerebrovascular findings, only two studies have been undertaken exclusively on patients aged over 60 years. Zhang et al. [28] included patients with an age range of between 60–74 years and found that those with OSA (mean AHI = 37) did not present any deterioration with respect to healthy controls in analytical variables that included haematocrit, blood viscosity, platelet aggregation, blood coagulability and fibrinogen levels. Hsu et al. [29], in their turn, did not observe any effect of CPAP treatment on sleepiness, quality of life or neurological recovery on 30 patients aged between 65–81 years with strokes and an AHI > 30, after randomisation to receive CPAP or conservative treatment for 8 weeks. In this case, however, the patients’ adherence to CPAP treatment was very poor (mean of 1.4 h/night) which can completely explain the results. Other studies that did not exclude elderly patients have produced inconsistent results. Some of them found drops in systolic and/or diastolic blood pressure after CPAP treatment but others did not observe any such effects. Three Spanish studies that did not exclude elderly patients and examined the effect of CPAP treatment on various parameters are particularly worthy of note here. Barbe et al. [30] and Durán et al. [31] found a drop of approximately 2 mmHg in blood-pressure readings, particularly in subjects with a good tolerance of CPAP, regardless of the symptoms. Parra et al. [32], however, did not observe any appreciable effect of CPAP treatment on functional recovery, quality of life or mortality after two years in a wide-ranging group of patients with a mean age of 64.7 (9.2) years at the acute phase of stroke. In any case, it must be stressed that an analysis of the distribution of the patients by age would be needed to adequately evaluate these results, especially with regards to the effect of CPAP on arterial hypertension and the subsequent impact on morbidity-mortality in elderly people.

It was not until 2014, however, that RCT focusing exclusively on clinical series of elderly patients (aged over 65–70 years) began to appear, in an attempt to clarify the role of CPAP treatment in this age group. Four studies stand out in this respect, and their general characteristics and key results are shown in Table 2.

The first of these studies, published by McMillan et al. [33], examined a cohort known as PREDICT (Continuous positive airway pressure in older people with obstructive sleep apnoea syndrome) in a 12-month, multi-centre, randomised trial undertaken in 14 centres in the UK. Two hundred and seventy-eight OSA patients aged 65 years or older (mean age 71.1 years) were randomised to receive CPAP or not, while they all received the best supportive care. A total of 231 patients completed the study (140 in the CPAP group and 138 in the non-CPAP group). OSA, diagnosed by means of a home polygraphic study, was defined as the presence of a desaturation index 4% greater than 7.5 events/hour plus an ESS value of more than nine points (mean ODI: 28.7; mean ESS: 11.6). The main variables were the ESS values at three months and the cost-effectiveness of the treatment assessed at 12 months, with secondary variables of quality of life, neurocognitive variables, clinical findings associated with OSA and cardiovascular risk factors in an intention-to-treat analysis. In summary, the patients who received CPAP treatment presented a clinically and statistically significant improvement in sleepiness of 2.1 (95% CI: −3, −1.3; *p* < 0.0001) after three months, and these results remained very similar after 12 months. The results were better in those patients with good compliance with CPAP or greater baseline sleepiness. CPAP also improved other clinical aspects, such as objective sleepiness, mobility and cholesterol readings after three months of treatment, although these results were not maintained at 12 months. Furthermore, no changes were observed in cardiovascular events’ neurocognitive variables, accident rates, nocturia or depression/anxiety. Finally, CPAP treatment did not prove to be a cost-effective treatment. One of the main limitations of this study was the generally poor adherence to CPAP treatment; even though most of the patients reported that they continued using it until the end of the study, the objective measurements revealed an average use of less than two hours after 12 months. 

The following year saw the publication of studies aimed more specifically at the sub-population of elderly OSA patients with a greater probability of improvement with CPAP, such as those with severe OSA. Dalmases et al. [34], performed a single-centre, pilot-study RCT on patients aged ≥ 65 years with severe OSA (AHI > 30), in which 33 patients were assigned to receive CPAP or sham-CPAP for three months. Those patients treated with CPAP presented a significant improvement with respect to the placebo group in episodic and short-term memory, executive function (speed of mental processing) and mental flexibility. Neuroimaging revealed an increased connectivity in the right middle frontal gyrus after three months of CPAP treatment and a higher percentage of cortical thinning in the conservative care group. No association was seen between cognition and brain functional connectivity changes within the default mode network.

Finally, the Spanish Sleep Group published the results of two clinical trials performed on a greater number of patients, and with a greater number of clinical outcomes, in patients aged over 70 years with severe and moderate OSA, respectively. In the first of these studies, Martinez-Garcia et al. [35] carried out a multi-centre study (in 12 clinical centres in Spain) that included 224 patients with a mean age of 75.5 years and an AHI ≥ 30 events per hour (mean AHI of 50.4 events/hour) referred to a sleep lab with suspicion of OSA. All the patients were randomised to receive CPAP (*n* = 115) or their usual control (*n* = 109) for three months. The diagnosis was made with either home polygraphy or polysomnography, depending on the possibilities of each centre. The main variable was the measurement of the quality of life via the Quebec Sleep Questionnaire (QSQ). The mean use of CPAP was 4.9 (SD: 2.5) hours/night, and 69.6% of the patients presented an adherence of more than 4 h/night. The CPAP group achieved a significant improvement (both clinically and statistically) in all the sleep-related symptoms analysed (snoring, witnessed apnoeas, nightmares, nocturia, asphyxic crises and sleepiness). In the latter case, specifically, the improvement, as measured by the ESS, was 3.38 points (CI 95% 2.5–4.2). Furthermore, there were improvements in all the domains of quality of life (QSQ), including sleepiness, daytime symptoms, night-time symptoms, emotions and social interaction. There was also a drop in the percentage of patients with abnormal scores in HADS tests (Hospital Anxiety and Depression Scale). As regards neurocognitive findings, an improvement was seen in working memory, but none were seen in other neurocognitive factors, such as visual attention and speed of processing, or in blood pressure readings after three months of treatment. 

Finally, the same group repeated this RCT in elderly patients with moderate OSA, replicating the methodology in order to provide comparisons. The only change was the main variable, which was now the ESS [36]. The patients were randomised to receive CPAP (*n* = 73) or no CPAP (*n* = 72) for three months. The mean age was 74.9 (4.6) years and the AHI ranged between 15–29.9 events/hour. The patients who received CPAP presented a drop in the ESS that was 2.6 (1.6–3.6) points more than that of the non-CPAP group. There were also improvements in some fields of the quality of life test (emotions and night-time symptoms) and in some sleep-related symptoms, but these improvements were less marked than those found in the patients with severe OSA. Moreover, in contrast with the severe OSA group, there were no identifiable improvements in neurocognitive findings in the test for anxiety and depression or in the blood-pressure readings.

### 4.2. Observational Studies

As in the case of RCT, there are very few observational studies that have exclusively, or almost exclusively, analysed the effect of CPAP on elderly people, and those that have done so mainly involve subgroups affected by diseases that have a particularly strong impact on the elderly, such as neurocognitive disorders and cerebrovascular disease. Aloia et al. [37] observed, in a small group of OSA patients (Respiratory disturbance index ≥ 10) aged over 55 years, that the use of CPAP for three months produced an improvement in neuropsychological parameters such as the capacity to concentrate and various psychomotor and verbal skills. Martínez-García et al. [38] observed, in their turn, that, although the impact of OSA on the quality of life in patients aged over 65 years was less than in younger people, treatment with CPAP significantly improved some aspects of the quality of life in both elderly and younger patients, when compared with the parameters of normality obtained in the general population of the same age and gender. This improvement was basically manifested in the findings related to sleepiness.

With regards to cardiovascular factors and beyond the intrinsic limitations of the observational studies, a particularly interesting retrospective study was conducted on 939 patients aged at least 65 years who were followed up for a mean of 69 months after being referred to a sleep specialist with suspicion of OSA. Those individuals with an AHI > 15 events/hour were adopted as a control group and those with an AHI > 30 events/hour were considered to have severe OSA. The severe cases that refused CPAP treatment or showed bad compliance presented excessive mortality from cardiovascular causes, and indeed from all other causes (especially stroke and heart failure) apart from coronary disease. However, in those patients who were treated with CPAP and showed good compliance (at least 4 h/night on average), this risk was normalised to levels similar to those of the patients in the control group. This excessive risk was not observed in patients with moderate OSA (AHI between 14 and 29), which supports the hypothesis that for an excessive risk, and therefore a positive effect from CPAP, to exist in elderly patients, the AHI needs to be higher than in younger individuals.

Furthermore, two studies carried out on an accumulated cohort of patients who had suffered a stroke (most of them elderly) and who were followed up for two and five years found that treatment with CPAP reduced, respectively, both the incidence of new cardiovascular events (especially a second stroke) and mortality, to the point of reaching levels similar to those of patients with a mild OSA, or none at all [39,40]. A recent observational study by López-Padilla et al. [41] on 155 individuals with an AHI ≥ 20 aged ≥ 80 years (the only study in the literature performed exclusively on such elderly patients) indicated better survival for those with good compliance with CPAP; these results are similar to those found by Ou et al. [42] in another recent study on a group of 130 patients with a mean age of 77.8 years. 

### 4.3. Compliance with CPAP

**Figure 1:**
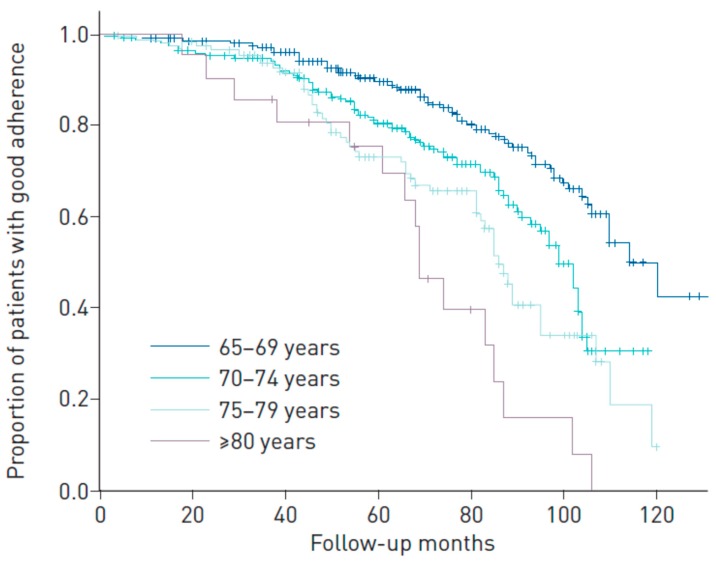
Kaplan-Meier curves according to age group for the percentage of patients with a CPAP compliance of at least four hours/day during follow-up. Martinez-Garcia et al. ERJ Open Res. 2019 Mar 4;5(1). pii: 00178-2018, with permission.

One important aspect of CPAP in elderly people is the level of compliance with the treatment. It seems be logical that an elderly patient could present a series of characteristics that have been associated with poorer compliance: living alone, fewer symptoms (particularly sleepiness), less dexterity, alterations in cognitive capacities, comorbidities, and neurological deficiencies [43,44]. However, the studies carried out in this respect offer discrepancies, especially in the elderly aged less than 80 years old. Martinez-Garcia et al. [45] observed a significant drop in adherence from the ages of 65 to 80 years: the mean (SD) CPAP use in hours/night was 5.2 (2.6), 4.6 (3); 3.8 (2.9) and 2.9 (1.7) for the respective age groups of 65–69, 70–74, 75–79 and >80 years (*p* for trend < 0.001). On average, therefore, those patients aged over 80 years presented an adherence of less than three hours, which is considered the limit of non-adherence (Figure 1). Moreover, in the clinical trial conducted by McMillan et al. [33], the degree of compliance with CPAP was very low after three months and, above all, after 12 months of treatment in patients aged over 65 years, even when, as demonstrated by Kostikas et al. [46], the observed pressure required for elderly patients is, on average, 2.5 mmHg less than that used for a younger people adjusted by OSA severity. 

## 5. Future Challenges

The study of OSA and its treatment with CPAP (and other alternative therapies) in the elderly is an authentic challenge, on both a clinical and scientific level. The number of elderly patients attending sleep units is gradually rising, and it is predicted to continue rising in the future. This situation contrasts with the limited data available on OSA in elderly people, as apparent in our fundamental ignorance about the cut-off point for the AHI that marks the limit, according to age, between its physiological and pathological manifestations. This gap in our knowledge makes the diagnosis of OSA and subsequent treatments especially difficult in this age group. It is possible that the AHI, as an objective variable, and the measurement of sleepiness especially by ESS, as a subjective variable, are not the most suitable indicators for a diagnosis of OSA in an elderly person. Table 3 presents some of the future challenges that need to be urgently tackled in order to further our understanding of OSA in these patients.

## 6. Conclusions

Although both basic research and complex, applied investigation are admirable and necessary, in OSA, as in other fields, we must continue to strive for answers to fundamental questions that have a direct impact on our daily clinical practice, such as: what is the appropriate diagnostic and therapeutic management of OSA in the elderly, and how do we achieve it? Answers to such questions are probably not that hard to find, as we have the raw data at hand, in the form of a large elderly population. Furthermore, the variables that need to be evaluated are fundamental to any clinical study: the impact of the disease, its diagnosis and the effect of treatment. The international guidelines on OSA point out some important factors that should be taken into consideration with respect to elderly patients. Firstly, a clinical history that includes findings specific to elderly people must be undertaken; secondly, sleepiness must not be considered a physiological symptom inherent to old age, as excessive daytime sleepiness is always pathological, regardless of the patient’s age; thirdly an excessive daytime hypersomnia should not assume to be always associated with OSA; and, finally, a patient with suspicion of OSA must not be denied any diagnostic or therapeutic procedures purely on the grounds of their age (Table 4).

## Figures and Tables

**Table 1:** Possible causes of greater airway collapsibility in an elderly person.

Increased Airway Resistance during Sleep
Reduction in the diameter of the pharynx due to fatty deposits on its walls
Pharyngeal muscular dysfunction
Alterations to the dilatory reflexes in the pharynx
Alterations to sleep structure
Greater respiratory instability during sleep (central events)
Post-menopausal women
Loss of teeth
Frequent comorbidities that are risk factors (stroke, heart failure, hypothyroidism, etc.)

**Table 2:** Characteristics and main results of the clinical trials undertaken on the effect of continuous positive airway pressure (CPAP) treatment in clinical series consisting exclusively of elderly patients (aged at least 65 years).

Study	Methodology	Allocation	Mean Age	Mean ESS	Mean BMI	Mean AHI	Follow-Up	Main Results
McMillan et al. (2014)	Multi-centre UK	140 CPAP 138 no CPAP	71.1 (4.6)	11.6 (3.7)	33.9 (5.7) 33.6 (6.4)	28.7 (19.1) *	12 months	Improvements in objective sleepiness, mobility, cholesterol levels and 2 points in ESS No changes in health-care costs, mood, functionality, nocturia, accidents, cognitive function and cardiovascular events
Dalmases et al. (2015)	Single-centre Spain	17 CPAP 16 sham CPAP	71.3 (5.5)	7.9 (3) 5.7 (3.6)	31.4 (4.3)	55.5 (17.6)	3 months	Improvements in episodic and short-term memory, executive functions, mental flexibility and neuronal connectivity (neuroimaging)
Martinez-Garcia et al. (2015)	Multi-centre Spain	115 CPAP 109 no CPAP	75.5 (3.9)	9.5 (3.8)	32.9 (6.3)	50.4 (14.9)	3 months	-Improvements in all QSQ domains, depression and anxiety indexes, sleep-related symptoms, working memory and 3.37 points in ESS No changes in blood pressure
Ponce et al. (2019)	Multi-centre Spain	73 CPAP 72 no CPAP	74.9 (4.6)	9.2 (4)	30.4 (5.5)	21.7 (4.8)	3 months	-Improvements in nocturnal symptoms and emotion domains (QSQ), sleep-related symptoms and 2.6 points in ESS No changes in nocturia, nightmares, QSQ domains except for nocturnal symptoms and emotions, anxiety and depression symptoms, and cognitive functions

* Oxygen desaturation index at 4%. Data are expressed as mean (standard deviation). ESS: Epworth Sleepiness Scale; BMI: Body mass Index; AHI: Apnoea-hypopnoea index. QSQ: Quebec Sleep Questionnaire.

**Table 3:** Future challenges in sleep apnoea in elderly patients.

Analysis of Changes in the AHI with Age and of the Best Parameters for Evaluating OSA in the Elderly
Validation of specific questionnaires for the elderly
Analysis of the clinical forms of the presentation of OSA in the elderly
Validation of simplified diagnostic tests
New devices adapted to the weaknesses of the elderly and frail patients
Treatment with CPAP in extreme old age (over 80 years)
Educational programmes aimed specifically at elderly people
Other therapeutic possibilities
Analysis of the consequences of the withdrawal of CPAP in extreme old age

**Table 4:** Some messages about obstructive sleep apnoea (OSA) and CPAP treatment in the elderly.

OSA Prevalence Increases with Age
It is not known what is the cut off in the apnea-hypopnea index and Epworth sleepiness scale that should be considered as pathological
A clinical history that includes findings specific to elderly people must be always undertaken
Usual sleepiness must not be considered a physiological situation in elderly. However it is not be assumed that it must be associated to OSA
The clinical picture of OSA in elderly is different than that seen in younger patients. Neurocognitive symptoms are especially prevalent
Full polysomnography is the gold standard in the diagnosis of OSA in elderly. However simplified test could be used in patients without important comorbidities
Randomized clinical trials demonstrated that CPAP is effective in symptomatic moderate to severe OSA patients
It is not known what happens in extreme old age (over 80 years) when the compliance to CPAP is very low
